# *In silico* design and validation of a novel multi-epitope vaccine candidate against structural proteins of Chikungunya virus using comprehensive immunoinformatics analyses

**DOI:** 10.1371/journal.pone.0285177

**Published:** 2023-05-05

**Authors:** Shirin Mahmoodi, Javad Zamani Amirzakaria, Abdolmajid Ghasemian

**Affiliations:** 1 Department of Medical Biotechnology, School of Medicine, Fasa University of Medical Sciences, Fasa, Iran; 2 Department of Plant Biotechnology, National Institute of Genetic Engineering and Biotechnology, Tehran, Iran; 3 Noncommunicable Diseases Research Center, Fasa University of Medical Sciences, Fasa, Iran; The 8th Medical Center of PLA General Hospital, CHINA

## Abstract

Chikungunya virus (CHIKV) is an emerging viral infectious agent with the potential of causing pandemic. There is neither a protective vaccine nor an approved drug against the virus. The aim of this study was design of a novel multi-epitope vaccine (MEV) candidate against the CHIKV structural proteins using comprehensive immunoinformatics and immune simulation analyses. In this study, using comprehensive immunoinformatics approaches, we developed a novel MEV candidate using the CHIKV structural proteins (E1, E2, 6 K, and E3). The polyprotein sequence was obtained from the UniProt Knowledgebase and saved in FASTA format. The helper and cytotoxic T lymphocytes (HTLs and CTLs respectively) and B cell epitopes were predicted. The toll-like receptor 4 (TLR4) agonist RS09 and PADRE epitope were employed as promising immunostimulatory adjuvant proteins. All vaccine components were fused using proper linkers. The MEV construct was checked in terms of antigenicity, allergenicity, immunogenicity, and physicochemical features. The docking of the MEV construct and the TLR4 and molecular dynamics (MD) simulation were also performed to assess the binding stability. The designed construct was non-allergen and was immunogen which efficiently stimulated immune responses using the proper synthetic adjuvant. The MEV candidate exhibited acceptable physicochemical features. Immune provocation included prediction of HTL, B cell, and CTL epitopes. The docking and MD simulation confirmed the stability of the docked TLR4-MEV complex. The high-level protein expression in the *Escherichia coli (E*. *coli)* host was observed through *in silico* cloning. The *in vitro*, *in vivo*, and clinical trial investigations are required to verify the findings of the current study.

## 1. Introduction

The Alphavirus genus, *Togaviridae* family, contains a positive and single-stranded RNA virus member known as Chikungunya virus (CHIKV) [1, 2]. The annual epidemic outbreaks of CHIKV occur in tropical and subtropical regions, where two mosquitoes including *Aedes aegypti* and *Aedes albopictus* play substantial roles as vectors of the virus [1, 3]. This agent has spread to various countries, posing clinical impacts such as fever, myalgia, headache, nausea, fatigue, and rashes in patients. The CHIKV is associated with severe influences on neonates, aged people, and vulnerable individuals [2]. The virus causes a series of self-limiting infections such as arthralgia and myalgia to more severe effects and even death among immunosuppressed and vulnerable individuals, in addition to considerable economic losses [2, 4]. CHIKV encodes various structural (E2, C, E1, 6K, E3) and non-structural (nsP1-4) proteins which can be matured and activated via proteolytic cleavage by viral or host enzymes [5–8]. The interactions of viral proteins such as E1 (fusion protein), E2 or p62 (glycoprotein adhesin), and temporary E2-binding protein E3 (unknown function) with the host cell surface initiate and develop the viral pathogenesis [8–10]. The E2 is the main target of the immune responses. The virus three different lineages have 92.5–98% identity at the amino acid sequence level [11, 12]. There is neither an efficient preventive [13] nor approved therapeutic approach to control the virus; hence seeking an appropriate vaccine is of utmost importance [13]. In the process of vaccine design, the mapping of INF-γ, B cells, and T cells using rapid and cost-effective immunoinformatics approaches is incredibly favorable. Considering these, a low-cost, safe and effective vaccine is promising to be applied in endemic countries with the CHIKV concerns. Various efforts aiming at the *in silico* designation of candidate vaccines utilizing structural and non-structural proteins, have demonstrated concedable results. However, some predictive tools need improvements in terms of sensitivity and specificity levels of prediction [14–21]. A vaccine efficacy is affected by immunogenicity, safety, thermostability and transportability factors. Subunit vaccines are preferable compared to whole inactivated or attenuated viruses owing to provocation of higher levels of specific host responses and lower adverse reactions or events [18, 21, 22].

The viral cognate receptors in specific tissues determine the tropism and infection. These receptors mostly include Prohibitin 1 (PHB1), T-cell immunoglobulin and mucin domain-1 (TIM-1), and Glycosaminoglycans (GAGs) [23]. Viral nsP2 inhibits the interferon-mediated JAK/STAT signaling [19]. CHIKV viral load remains detectable up to more than 35 weeks post-infection [24, 25]. The viral RNA causes inflammatory and chronic diseases mainly driven by IFN-α [26]. Although several antivirals have been applied, genetic mutations and the development of resistance have occurred. The CHIKV manifestations may be mistaken for Zika or dengue fever with lower mortality rates, thus exact laboratory diagnosis is crucial [27]. Among various vaccines, inactivated or killed vaccines has high production cost impeding its accessibility [22]. Live attenuated virus provokes higher immune responses compared to inactive vaccines. Subunit vaccines depend on the adjuvant used due to the various efficacy levels of adjuvants [28]. A suitable adjuvant should contribute to the decrease of multiple doses of subjection and provide long-lived immunity and protection from viremia [29]. It has been also revealed that low-molecular weight or protein-based adjuvants enhance the neutralization ability and antibody titers [30]. The RS09; (sequence: APPHALS) is the TLR4 agonist which causes co-stimulation of CTL epitopes. This synthetic adjuvant provides higher safety as a potential immunogenic protein compared to other traditional adjuvants [31–33]. The objective of the current survey was the design a novel multi-epitope vaccine (MEV) candidate against the CHIKV using immunoinformatics analyses.

## 2. Methodology

### 2.1. Retrieval of proteins sequences

The polyprotein (E1, E2, 6 K, and E3) structure of the Chikungunya virus (CHIKV) with accession number Q1H8W5 was targeted for epitopes prediction which was retrieved from the UniProt protein database (https://www.uniprot.org/) and then saved in FASTA format for additional analyses.

### 2.2. Immuno-informatics analyses

#### 2.2.1. Prediction of helper T cell epitopes

Immune Epitope Database (IEDB) (http://www.iedb.org/) was utilized to predict helper and cytotoxic T lymphocytes (HTLs and CTLs, respectively) of the CHIKV structural proteins. The IEDB applies various prediction methods, containing consensus method, Sturniolo method, stabilized matrix method (SMM)-align, and average relative binding (ARB) for MHC-II binding epitopes prediction [34].

#### 2.2.2. Prediction of cytotoxic T cell epitopes

CTLs play a central role in the immune system response to intracellular infections. They distinguish defective cells by binding to presented peptides on the cell surface by MHC class I molecules. Therefore using the NetMHCpan-4.1 server (https://services.healthtech.dtu.dk/service.php?NetMHCpan-4.1), binding CTL epitopes of the CHIKV structural polyprotein to four mouse alleles (Db, Dd, KK and Kb) were predicted. The NetMHCpan-4.1 server predicts the binding of peptides to any MHC molecule of the known sequence using artificial neural networks (ANNs) [35].

#### 2.2.3. Continuous B-cell epitopes prediction

B-cell epitopes have a vital role in the designing of protein vaccines, hence using ABCpred server continuous B-cell epitopes were selected from the CHIKV structural polyprotein. This server predicts continuous B cell epitopes based on artificial neural network and recurrent neural network (machine-based technique) through fixed length patterns with an accuracy of 65.93%.

### 2.3. Vaccine construct designing

The multi-subunit sequence contained an HTL epitope followed by three B cell epitopes and two CTL epitopes regions. To improve the MEV construct efficacy, the GPGPG and KK linkers were used for epitopes connection [36, 37]. Additionally, the designed vaccine construct contained the universal T helper epitopes, PADRE (Pan HLA-DR reactive epitope) linked with the C-terminal region of the MEV candidate via an EAAAK linker and the TLR-4 agonist (RS09; Sequence: APPHALS) as an adjuvant joined via KK linker to the vaccine construct for increasing the immune responses [38, 39].

### 2.4. Structural analysis of the designed vaccine construct

The antigenicity prediction of the designed MEV construct was performed using ANTIGENpro and Vaxijen v2.0 servers. ANTIGENpro is an alignment-free software that predicts antigenicity according to the obtained results by protein microarray data analysis. Vaxijen applies a promising alignment-independent method based on a protein sequence mining technique [40]. Additionally, the allergenicity of the MEV construct was predicted using the AllergenFP server, implemented based on the physicochemical properties of proteins with approximately 88% accuracy [41]. The physicochemical properties of designed vaccine, including molecular weight (Mw), theoretical pI, instability index, half-life, the total number of positive and negative residues, and grand average of hydropathicity (GRAVY) were predicted using the ProtParam tool (http://web.expasy.org/protparam) [42]. The secondary structure of the MEV construct was predicted using the PSIPRED V3.3 server (http://bioinf.cs.ucl.Ac.uk/psipredtest) [43].

### 2.5. 3D modeling of the vaccine construct and model refinement

I-TASSER online server at https://zhanggroup.org/I-TASSER/ was applied to predict the 3D structure of the MEV candidate. The quality of the structure was evaluated using Z-score and Ramachandran plot through ProSa [44] and PROCHECK [45] servers. The protein was further refined using side-chain minimization at a maximum of steps per residue (10000) using Molegro Virtual Docker (MVD). The refined structure was used for subsequent analysis.

Furthermore, the stability of the predicted model was evaluated through molecular dynamic (MD) simulation as the procedure described in next section.

### 2.6. Molecular docking of vaccine construct with TLR4 receptor

The TLR4 (PDB code: 4G8A) 3D structure was retrieved from RCSB Protein Data Bank (RCSB PDB) (https://www.rcsb.org/) and subjected to the PyMOL v2.3.4 software for energy correction. The water molecules were removed from the PDB structure. TLR4 and MEV candidate 3D models were submitted to the HDOCK server (http://hdock.phys.hust.edu.cn/) [46] to assess the interaction of TLR4 and vaccine. According to the results, the highest-ranking complex was selected at the lowest intermolecular binding energy between the vaccine and the TLR4.

### 2.7. Molecular dynamic simulation

The MD simulation was applied to evaluate the stability of the vaccine-TLR4 complex result from docking simulation using GROMACS 2018. The structure was placed in a dodecahedron box and filled with water using the tip3 water model. To neutralize systems some molecules of water were randomly replaced by Cl^-^ or Na^+^. After neutralization, the steepest descent algorithm was used for energy minimization. The system Equilibration was conducted under 100 ps NVT at the temperature of 298 K followed by 100 ps NPT ensembles at the pressure of 1 bar. Electrostatic interactions were calculated by PME [47] and constrain of all bonds connecting hydrogen atoms was performed using the LINCS procedure. The Final MD simulation was run for 100 ns with no restraint. The binding energy was calculated using Molecular mechanics/Poisson–Boltzmann (Generalized-Born) method using gmxMMPBSA tools [48].

### 2.8. Discontinuous B-cell epitopes prediction

The predicted 3D model of the vaccine construct was used as an input file for ElliPro server subjection to determine the discontinuous B cell epitopes. The ElliPro tool predicts antigenic protein residues via Thornton’s method, using a protein 3D model [49].

### 2.9. *In Silico* cloning and mRNA secondary structure prediction

To convert the amino acid sequence to nucleotide sequence, reverse translation was performed using the Jcat tool (http://www.prodoric.de/JCat). Then, GenScript Rare Codon Analysis Tool (https://www.genscript.com/tools/rare-codon-analysis) was utilized to optimize the DNA sequence for cloning and expression into *the E*. *coli* host. Finally, *Nde*I and *Bam*HI restriction sites were added to the N and C-terminal regions of the gene sequence, respectively to clone the adapted MEV construct DNA sequence into the *E*. *coli* pET-14b vector. Then, the RNAfold tool of ViennaRNA Package 2.0 (http://rna.tbi.univie.ac.at/cgi-bin/RNAWebSuite/RNAfold.cgi) was applied to predict the secondary structure of the mRNA sequence. The server predicts the mRNA structures thermodynamically and assigns a minimal free energy score (MFE). Hence, the MFE structure and the centroid secondary structure, as well as their minimum free energy were calculated [50].

### 2.10. Immune simulation

Immune response stimulation by employing the C-ImmSim server (http://kraken.iac.rm.cnr.it/C-IMMSIM/) evaluated the efficiency of the designed vaccine *in silico*. This server applies a position-specific scoring matrix (PSSM) to identify immunological epitopes and immune interactions [51]. Three peptide vaccine injections were given for 350 days at time steps 0, 120, and 240. The simulation volume of the vaccine (containing no LPS) injection was set at 10. Random seed and time step were set at 234 and 1024 respectively.

## 3. Results

### 3.1. Immunoinformatics analyses

#### 3.1.1. Prediction of HTL, CTL, and B cell epitopes

Those top-scoring HTL (I-Ab, I-Ad, and I-Ed) and CTL (Db, Dd, Kb, and KK) alleles were predicted from the CHIKV structural polyprotein (E1, E2, 6 K, and E3) using IEDB and NetMHCpan servers respectively. In addition, the continuous B-cell epitopes were identified from the CHIKV structural polyprotein using ABCpred server (Table 1).

**Table 1:** Predicted HTL, CTL and continuous B cell epitopes of the CHIKV structural polyprotein.

HTL epitopes	CTL epitopes	Continuous B cell epitopes
_23_PTIQVIRPRPRPQRQAGQLAQLI_45_	_354_HSPVALERI_362_	_122_KvTGYACLVGDKVMKP_137_
	_725_YELTPGATVPFLLSLI_740_	_673_HGHPEIILYYYELYP_689_
		_992_DVYNMDYPPFGAGRPG_1007_

HTL: helper T lymphocytes, CTL: cytotoxic T lymphocytes

### 3.2. Devising potential multi-epitope vaccine candidate

**Fig 1:**
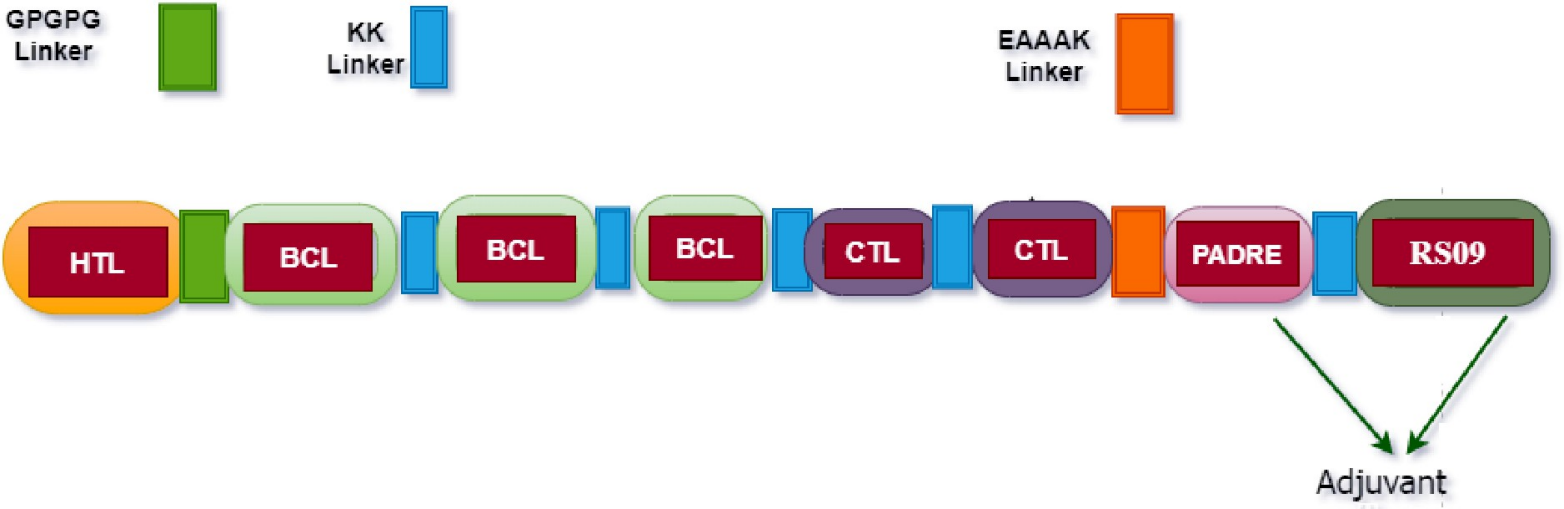
The diagram of the designed MEV construct composed of helper T lymphocytes (HTL), B cell (BCL), cytotoxic T lymphocytes (CTL) epitopes and adjuvant joined together using proper linkers.

The designed MEV candidate construct included one HTL, three B-cell, two CTL epitopes and the adjuvant (PADRE and RS09- TLR-4 agonist). The MEV construct components were fused using proper linkers (GPGPG, KK, and EAAAK) (Fig 1).

### 3.3. Designed vaccine construct evaluation

**Fig 2:**
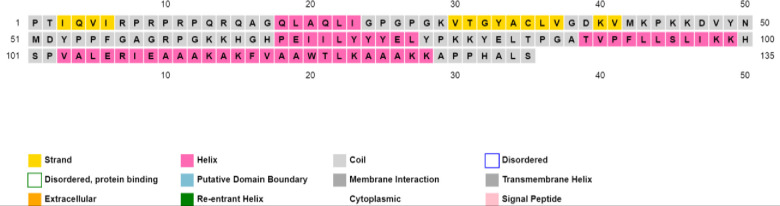
PSIPRED graphical results from secondary structure estimation of the designed vaccine construct.

The designed MEV construct was evaluated for its functional properties. The antigenicity (using the ANTIGENpro server) included 0.8 and (using the VaxiJen 2.0 server) included 0.65 by a virus model at a threshold of 0.4. Additionally, the MEV construct was predicted to be non-allergenic by the AllergenFP server. The physicochemical properties of the MEV construct were predicted by the ProtParam server. The molecular weight included 14.795 kDa with 135 amino acids, aliphatic index of 84.67, pI value of 10 (indicating stability in nature) and GRAVY score of -0.326. Additionally, an instability index of 35.03 predicted the protein vaccine stability. Total number of positively and negatively charged residues included 22 and 8, respectively. The estimated half-life was calculated as > 20 hours in mammalian reticulocytes, *in vitro* and >20 hours in yeast *in vivo*. Moreover, the secondary structure of the designed MEV construct was determined using the PSIPRED V3.3 server, where 10.37% of the sequence consisted of the strand, 50.37% consisted of coil and the remaining 39.25% composed of helix structure (Fig 2).

### 3.4. 3D modeling of vaccine construct and model refinement

**Fig 3:**
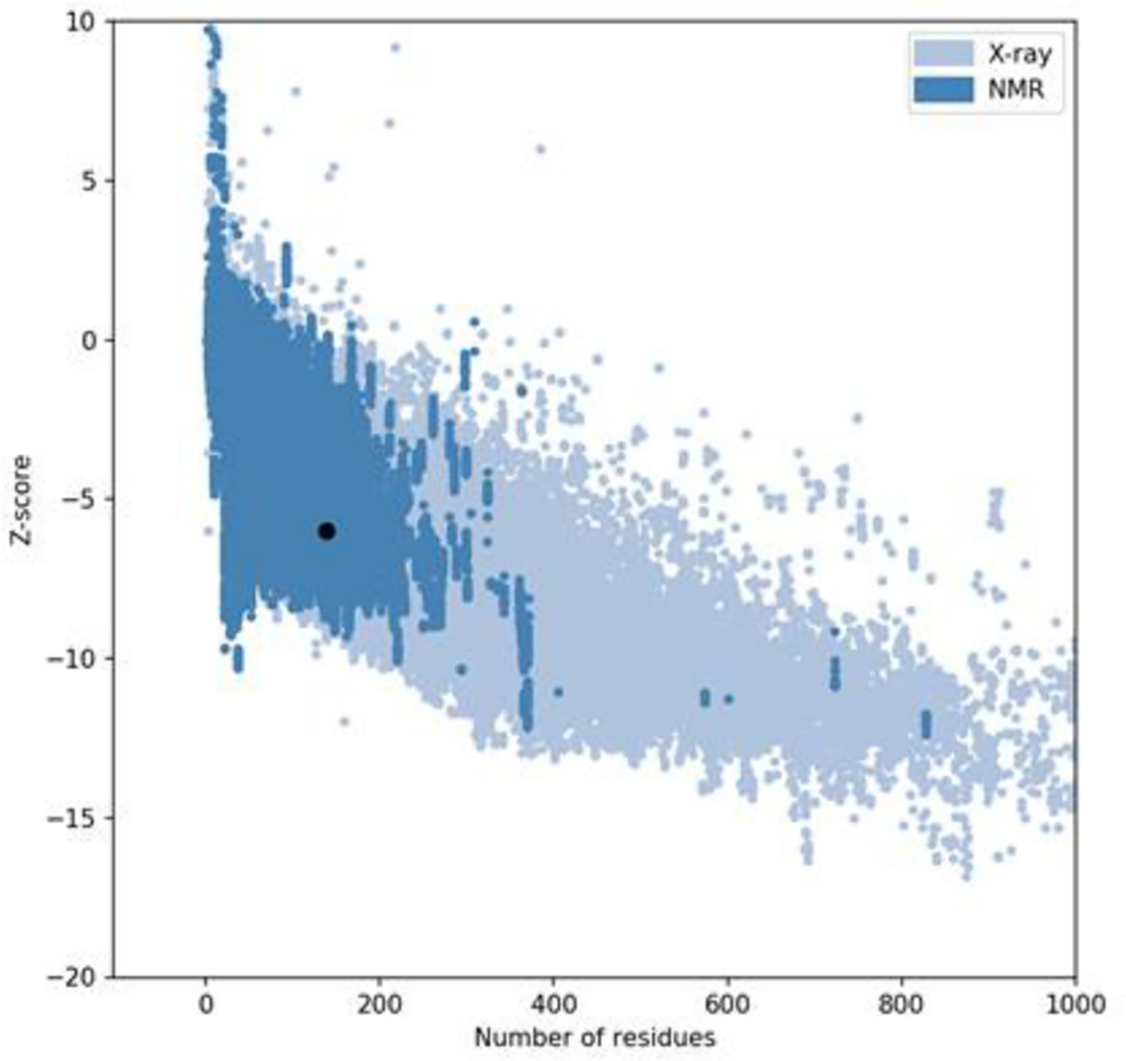
Validation analysis of the MEV 3D structure by ProSA-web server exhibiting the z-score in the range of native protein conformation.

**Fig 4:**
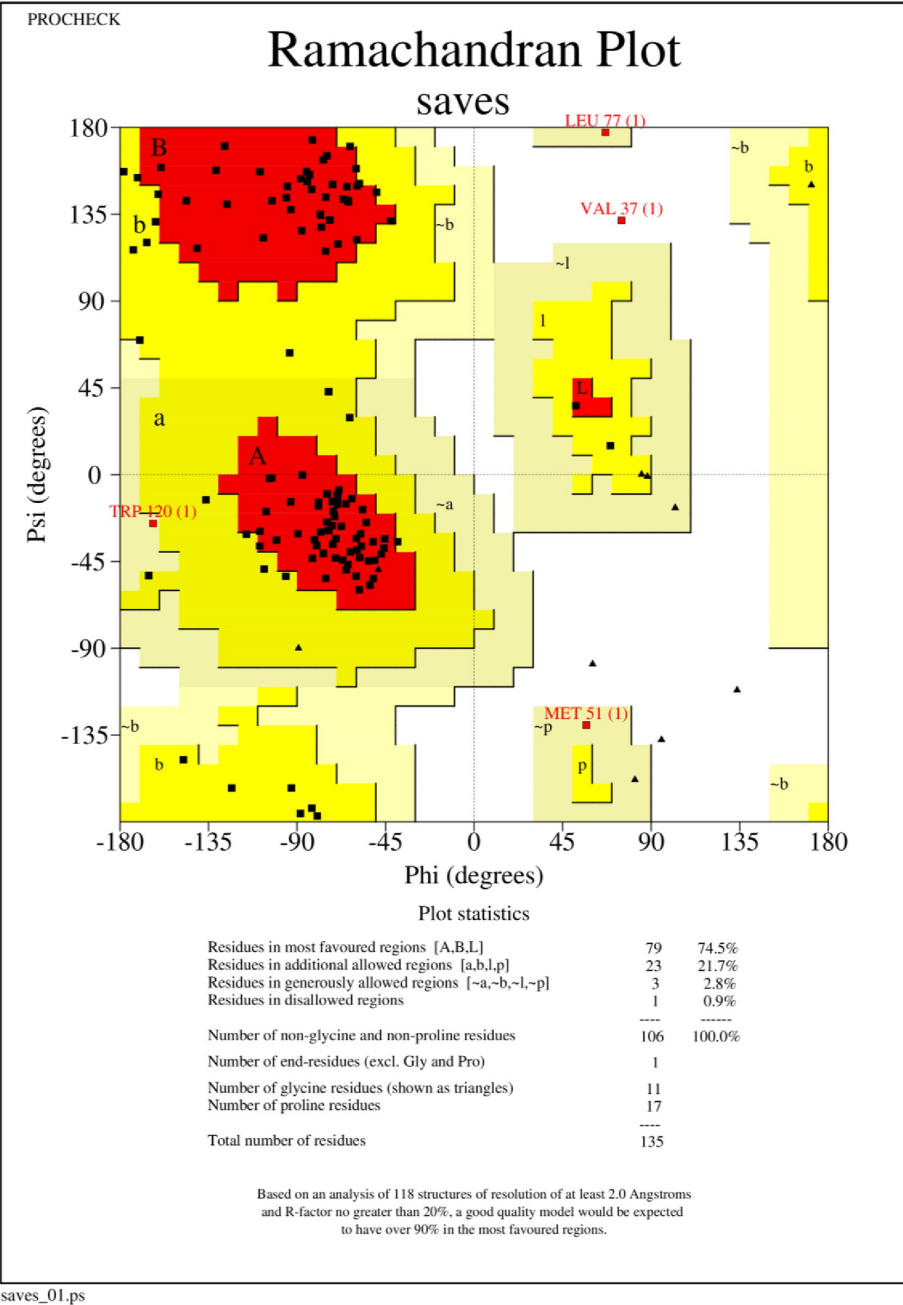
The ramachandran plot exhibited that 99.1 percent of residues were within the most favored allowed regions.

The 3D structure of the MEV was modeled using the I-TASSER server (S1 Fig) and its structure was validated using Z-score and Ramachandran plots. The z-score of the model was within the range of scores typically found for native proteins of similar size (Fig 3). The ramachandran plot showed that 99.1 percent of residues were within the most favored in allowed regions (Fig 4).

### 3.5. Docking of the vaccine construct with TLR4

**Fig 5:**
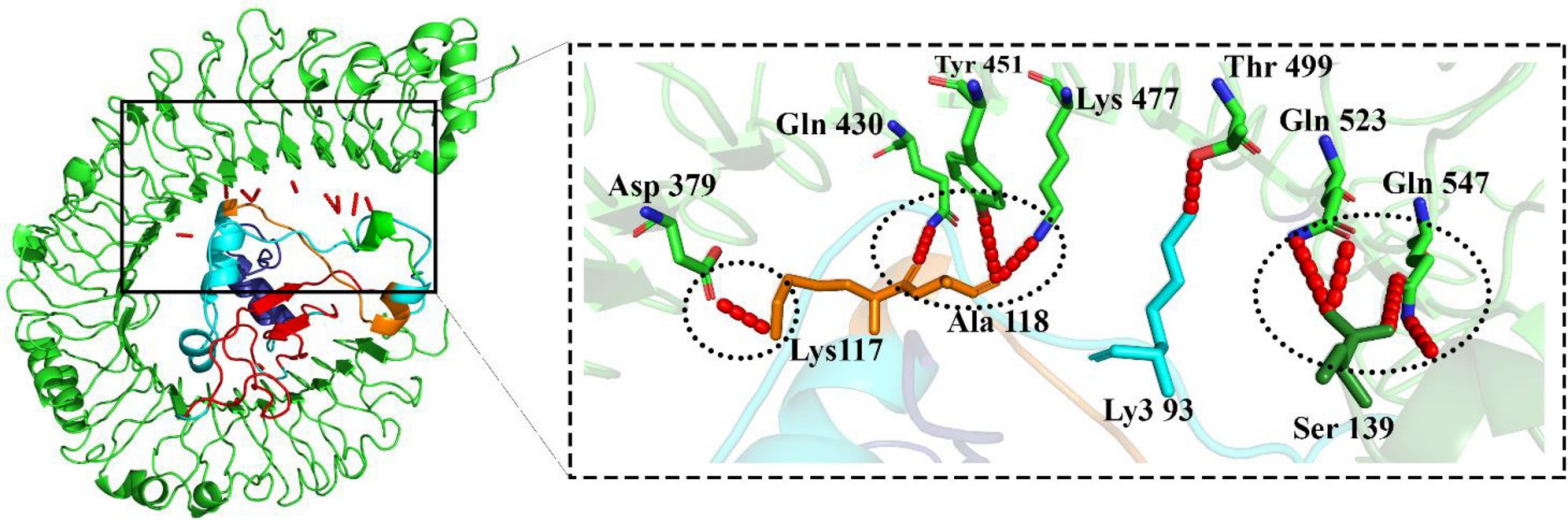
The MEV model in complex with the TLR4; dark green and orange: Adjuvant, dark blue: Helper T lymphocyte epitopes, red: B cell epitopes, blue: Cytotoxic T lymphocyte epitopes, green: TLR4 and red: Hydrogen bonds.

**Fig 6:**
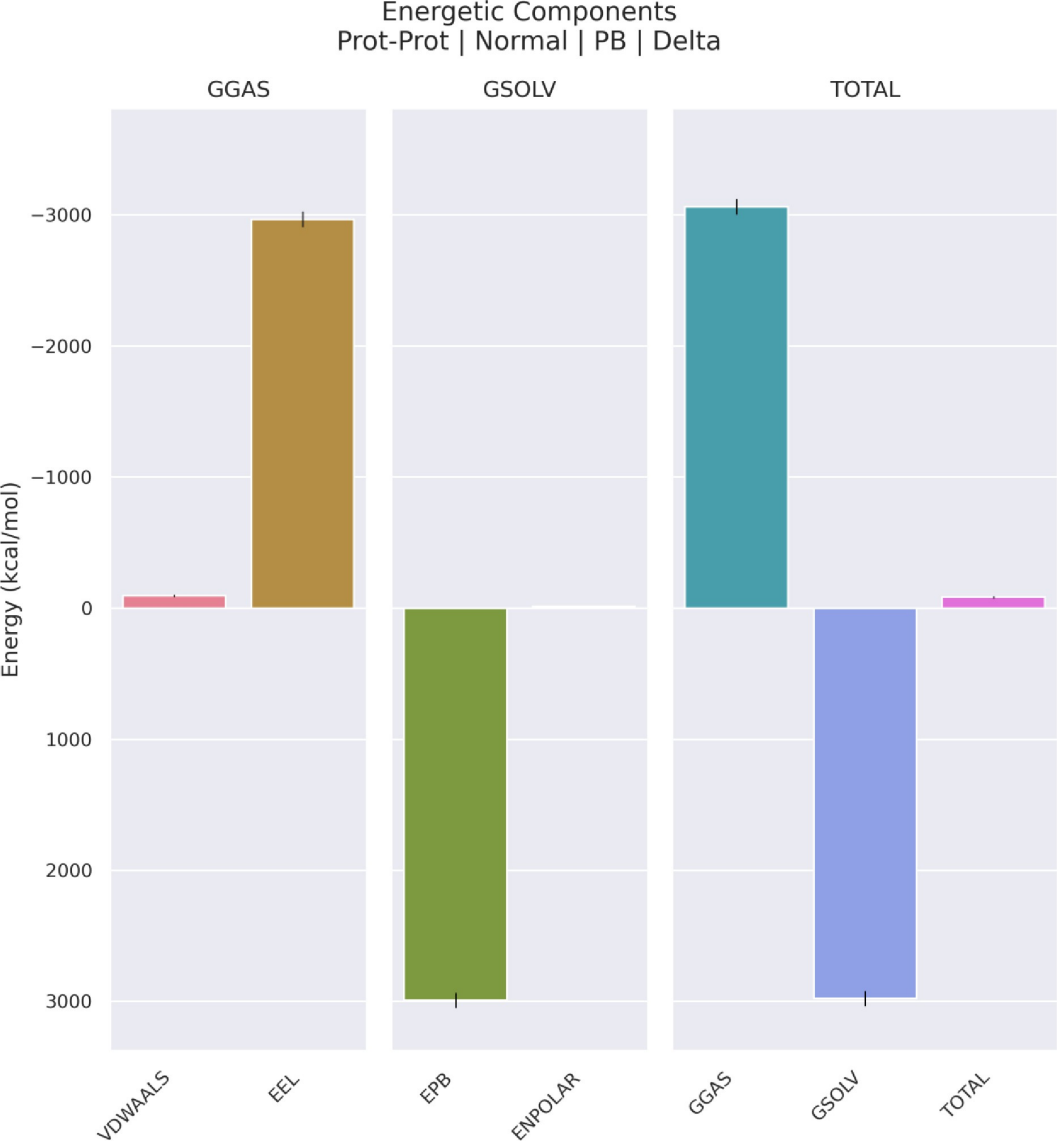
The different components of binding energy between TLR4 and vaccine calculated by gmxMMPBSA tool.

Docking results exhibited that the MEV construct interacted with the TLR4 3D structure via several amino acids of the TLR4 (Fig 5). The free energy of binding between the MEV and TLR4 during the MD simulations was calculated by applying the ΔGMMPBSA. The calculated score included -84.66 Kcal/mol (Fig 6).

### 3.6. Molecular dynamic simulation

#### 3.6.1. Root Mean Square deviation (RMSD)

**Fig 7:**
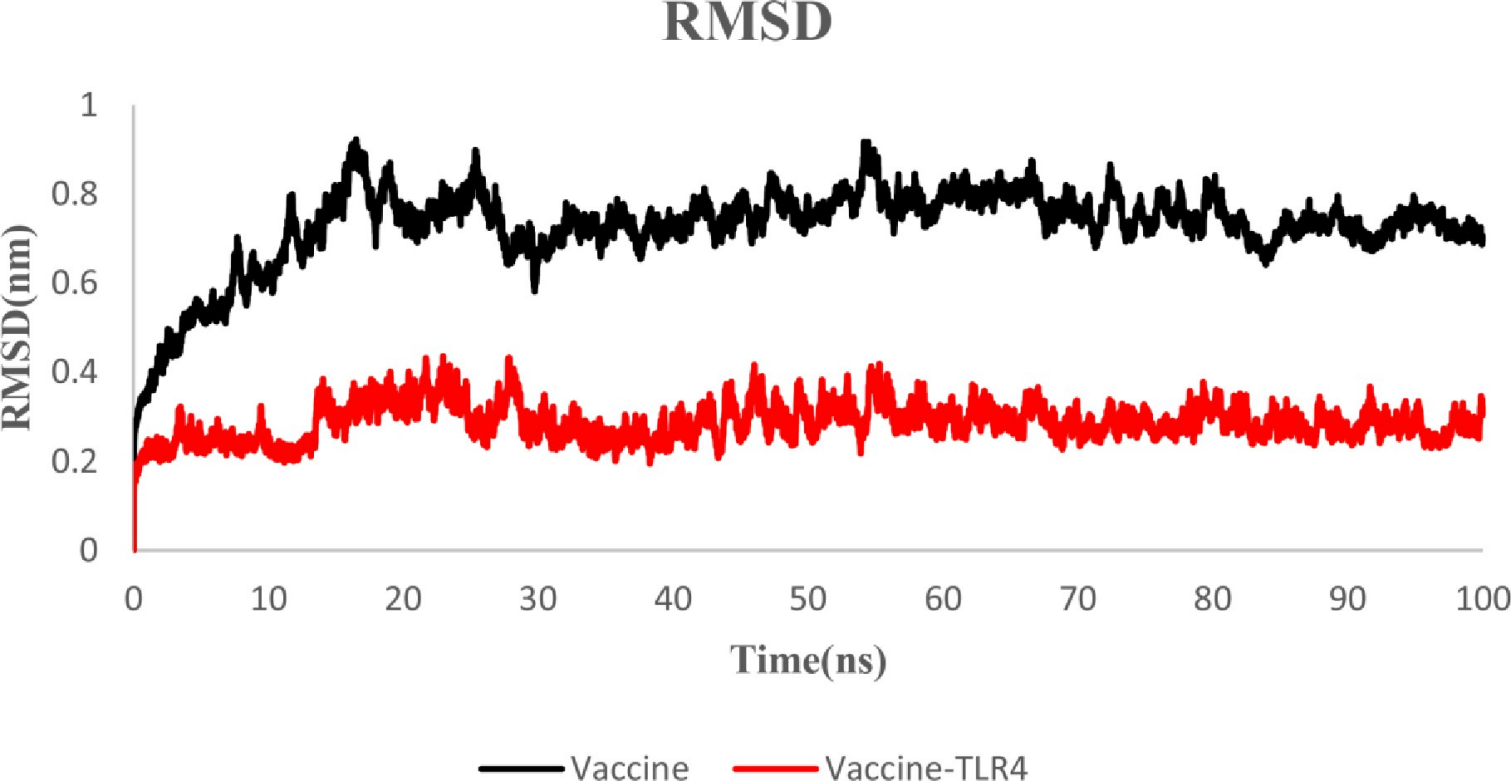
RMSD plot of the MEV and MEV-TLR4 complex: Black: Vaccine, red: MEV-TLR4 complex.

The stability of the MEV and MEV-TLR4 complex was evaluated using the Root Mean Square deviation (RMSD) of the backbone atoms. This plot indicates the protein conformational alterations during MD simulation from the initial structure. The MEV construct 3D structure confirmed that the RMSD values were in the range of 0.2 to 0.75 nm in free form and 0.2 to 0.5 nm in docked form. After 50 ns of simulation, both structures reached stability. According to the RMSD plot, both structures were stable. However, the MEV-TLR4 complex was more stable than the MEV construct free form (Figs 6 and 7).

#### 3.6.2. The root-mean-square fluctuation (RMSF)

**Fig 8:**
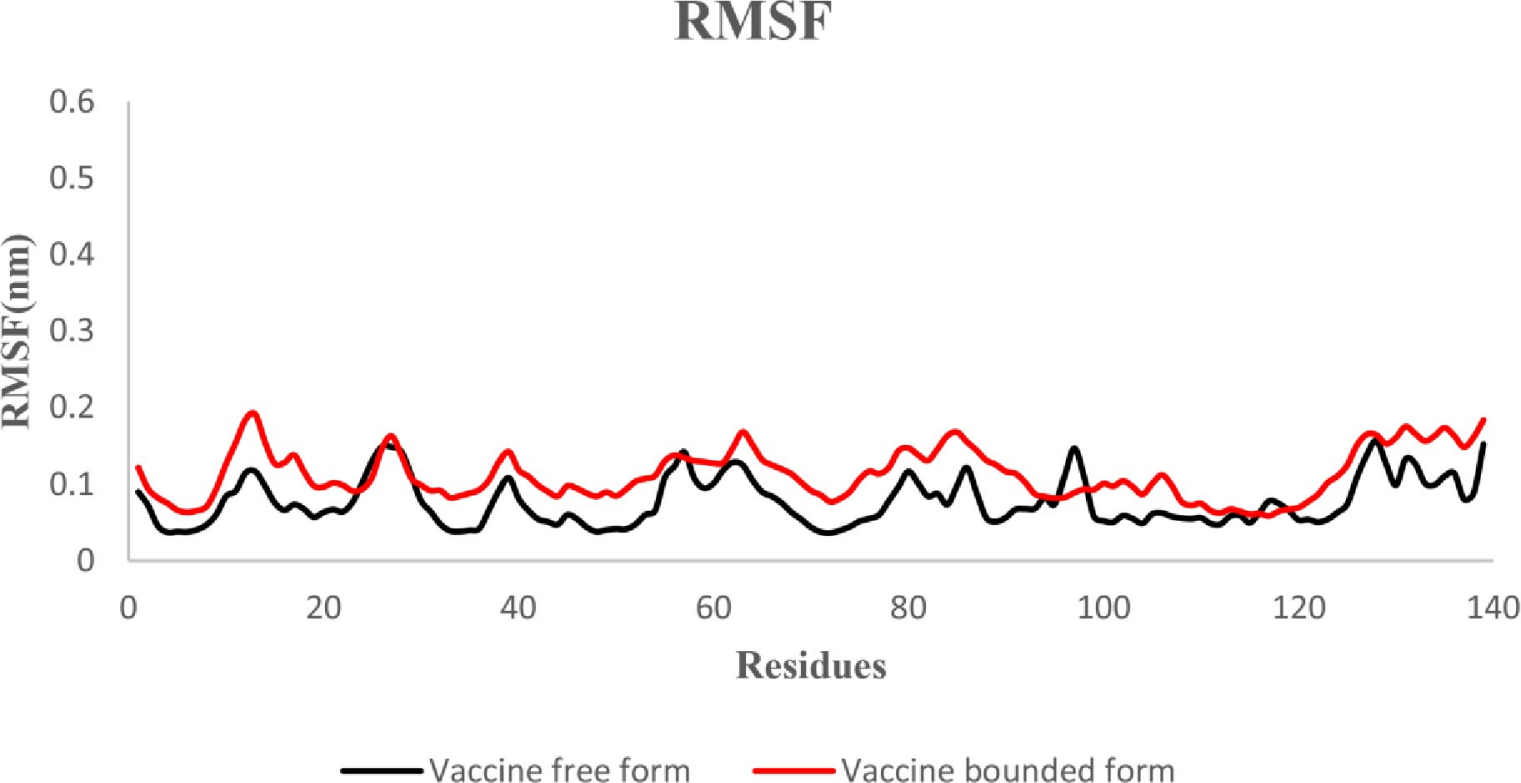
RMSF plot of the vaccine in the free and docked state: Black: Free-state, red: Docked state.

The root-mean-square fluctuation (RMSF) indicates the fluctuation of protein residues over time from a reference position during simulation. Here we evaluated the fluctuation of the MEV construct in the free-state and in the docked complex. According to the results, no unusual fluctuation was observed in the both states of the MEV candidate construct (Fig 8).

#### 3.6.3. Radius of gyration

**Fig 9:**
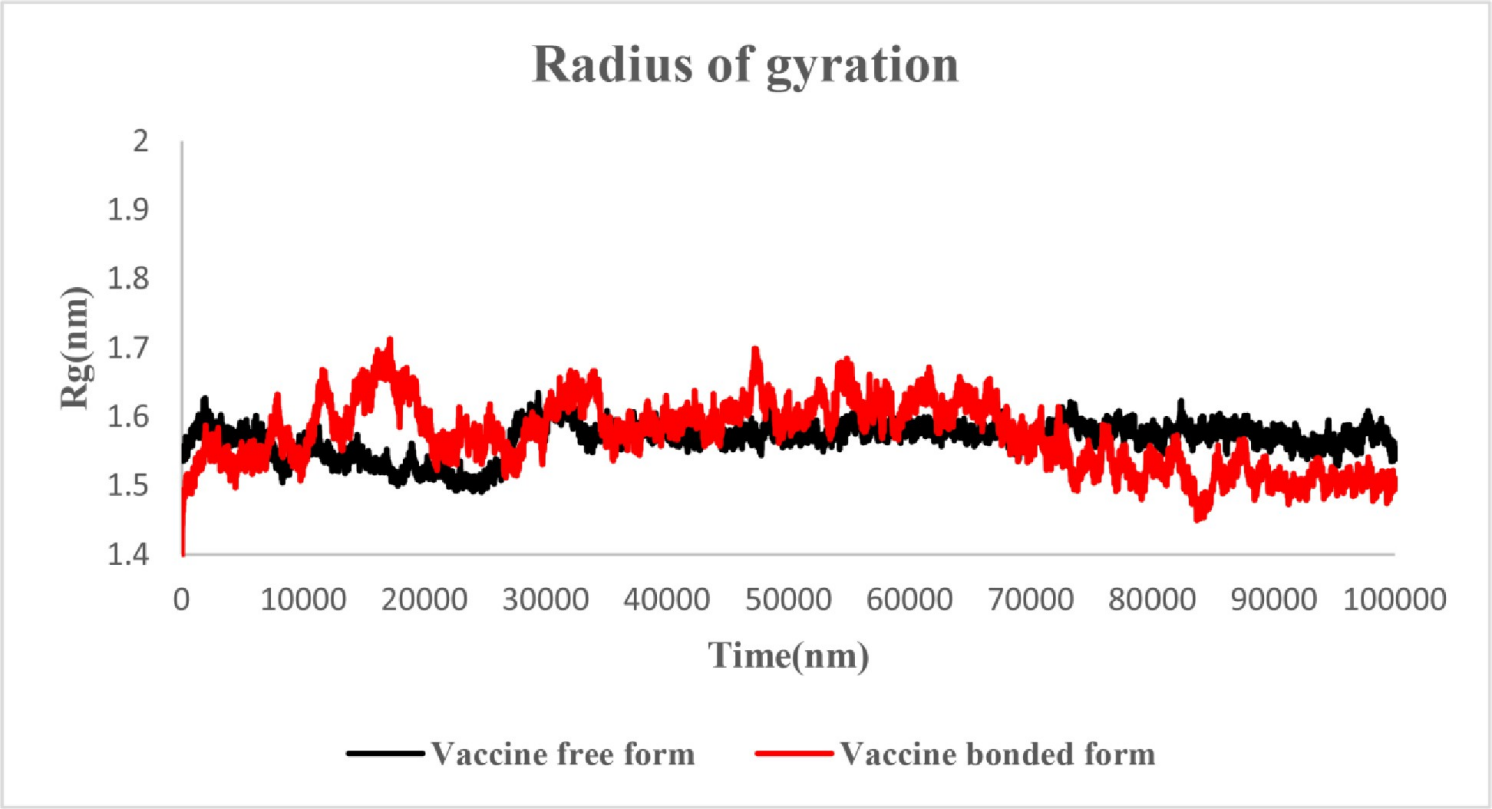
Rg plot of the MEV in free and docked states; black: Free-state, red: Docked state hydrogen bonds.

We evaluated the change in compactness of the MEV during simulation using Radius of gyration (Rg) plot. According to the results, the Rg of free and docked state were in the range of 1.45 to 1.65. After 65 ns, the Rg of both structures reached stability and remained stable (Fig 9).

#### 3.6.4. Hydrogen bonds

To evaluate the MEV-TLR4 complex, the number of hydrogen bonds between the MEV construct and the TLR4 was calculated (S2 Fig). Furthermore, the distribution probability of hydrogen bonds was plotted. According to this plot, six hydrogen bonds were formed between the MEV candidate and the TLR4 with higher probability during simulation (S3 Fig).

#### 3.6.5. dssp analysis

To evaluate the change in secondary structure during simulations, dssp analysis was performed. The percentage of secondary structures in the free and docked state of the MEV candidate was calculated. No significant difference was observed in the secondary structure of the MEV candidate in neither free nor bounded form. Accordingly, the results revealed the stability of both states of the MEV construct (S4 Fig).

### 3.7. Discontinuous B-cell epitopes prediction

Discontinuous B-Cell epitopes were predicted from the 3D structure of the MEV construct using the ElliPro server (Table 2).

**Table 2:** Conformational B-cell epitopes from 3D MEV construct protein predicted by ElliPro server.

Discontinuous B cell epitopes	Number of residues	Score
1:P1, 1:T2, 1:I3, 1:Q4, 1:V5, 1:I6, 1:R7, 1:P8, 1:P10, 1:Q15, 1:Q21	14	0.769
1:F56, 1:G57, 1:A58, 1:G59, 1:R60, 1:P61, 1:G62, 1:K63, 1:K64, 1:H65, 1:G66, 1:H67, 1:P68, 1:E69, 1:I70, 1:I71, 1:Y82, 1:E83, 1:L84, 1:T85, 1:P86, 1:G87, 1:A125, 1:A126, 1:K127, 1:K128, 1:A129, 1:P130, 1:P131, 1:H132, 1:A133, 1:L134, 1:S135	33	0.681
1:D39, 1:K40, 1:V41, 1:K43, 1:P44, 1:K45, 1:K46, 1:D47, 1:V48, 1:H100, 1:S101, 1:P102, 1:V103, 1:R107	14	0.674
1:A110, 1:A112, 1:K113, 1:A114, 1:K115	5	0.604
1:R9, 1:K29, 1:G32, 1:Y33, 1:A34	5	0.519

### 3.8. *In Silico* cloning and mRNA secondary structure

Reverse translation and codon optimization of the designed MEV construct were performed. The significant properties of the gene sequence to achieve a high-level protein expression in the *E*. *coli* host, including Codon Adaptation Index (CAI), GC content, and Codon Frequency Distribution (CFD), were estimated by the GenScript Tool. The CAI of the optimized nucleotide sequence was 0.85. The average GC content of the MEV sequence was 52.36. The results of 100% CFD value were obtained for the sequence (S5A–S5C Fig). The secondary structures of the mRNA along with their corresponding free energies were evaluated using the RNAfold server. The MFE secondary structure had a minimum free energy of -126.50 kcal/mol, while that of centroid secondary structure included -106.80 kcal/mol. These results suggest that the mRNA could remain stable after manufacturing (S5D and S5E Fig).

### 3.9. Immune stimulation

**Fig 10:**
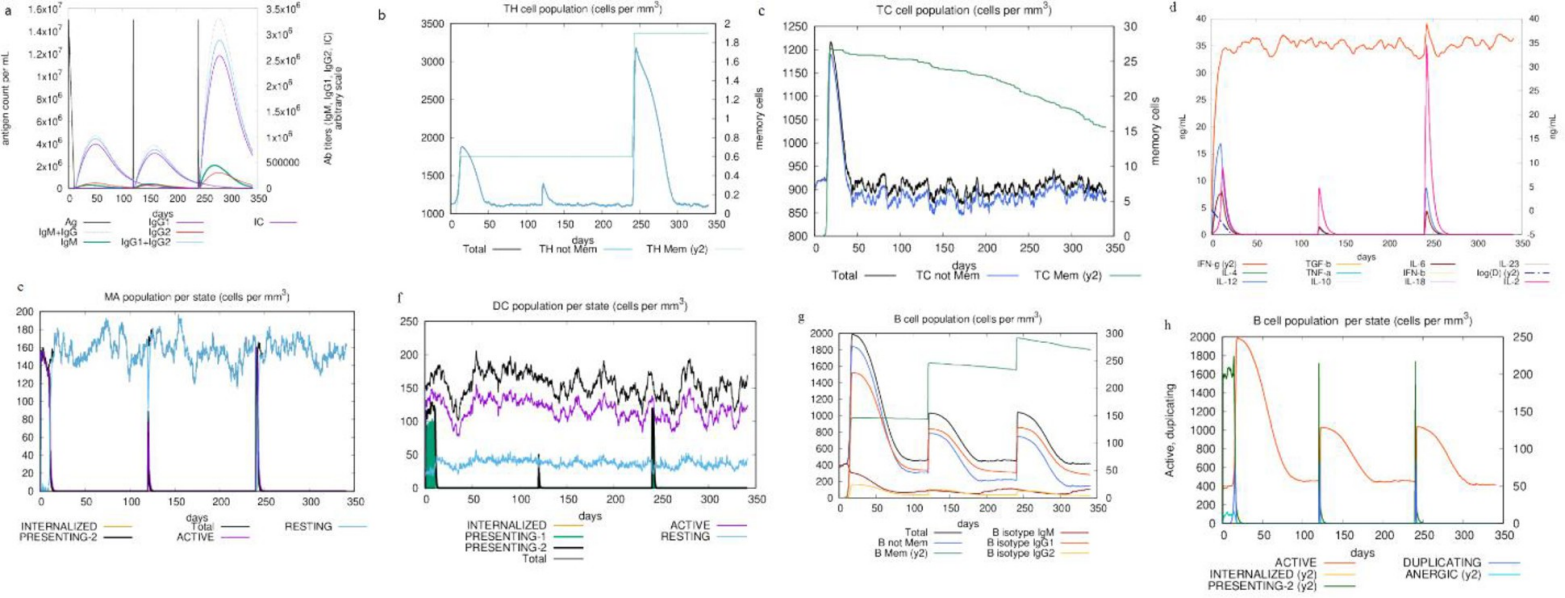
Immune simulation of the MEV candidate, a: immunoglobulin production on subsequent injection of antigens (shown by black lines); colored lines indicative of immune cells class, b: production of HTLs or helper T lymphocytes. c: production of CTLs or cytotoxic T lymphocytes, d: increase of cytokines and interleukins release for effective immune response. e: macrophages population, f: dendritic cells population, g: B-cell population, h: B-cell population per state.

The C-ImmSim server was used for the immune simulation. This reveals an immunological response similar to the body immune response. An enhancement in IgM+IgG levels was characteristic of the first reaction, followed by increases in levels of IgM and IgG1+IgG2, respectively (Fig 10A). In addition, the findings demonstrated the formation of memory cells and increase in the number of HTLs (Fig 10B), CTLs (Fig 10C), cytokines (IFN-γ and IL-2) (Fig 10D), macrophages (Fig 10E) and dendritic cells (DCs) after further exposure (Fig 10F). Both the secondary and tertiary stages of the immune responses were distinguished by the presence of a significant number of B-cells population (Fig 10G and 10H).

## 4. Discussion

The CHIKV causes Chikungunya fever (CHIKF) which is a health problem worldwide and a recurrent infection for which societies suffer from a lack of efficient preventive or therapeutic approaches [52, 53]. Vulnerable individuals such as children and those >60 years are more susceptible to the disease. Various vaccine candidate platforms such as live attenuated vaccine (LAV), viral vector, chimeric virus, virus-like particle (VLP), and DNA vaccines have been introduced against CHIKV.

Bioinformatics tools can predict various aspects (structural and physicochemical characters) of biomolecular structures such as proteins/epitopes and their interactions with the targets. The advantage of these approaches includes predicting rapid, valid, and inexpensive basic results [54, 55].

The development of subunit MEVs has advantages such as proper immunity provocation, safety (due to specific epitopes), and protection without allergic reactions or acute viremia. The virus structural and non-structural proteins such as capsid, E1, E2, E3, P62, 6K, Nsp1, Nsp2, Nsp3, and Nsp4 have been utilized in the MEV candidate constructs. These proteins act in viral binding and assembly and immune evasion or JAK-STAT signaling, INF-β and the NF-κB promoter inhibition [15, 19, 56–58]. E1 and E2-based subunit vaccines are strongly adjuvant-dependent and have elicited antibody responses in AG129 mice [21, 59]. An MEV candidate can stimulate more powerful immune responses than a single protein. Notably, the immunogenicity of vaccines can be enhanced using adjuvants that elicit humoral and cell-mediated responses [60]. Clinical trials of subunit vaccines have revealed their potential for antibody response stimulation [61]. E1 and E2 proteins fused to the Fc of IgM could elicit antibody responses and a balance of Th1/Th2 activation in C57BL/6 mice [62]. The immunization of BALB/c mice with CHIKV full-length E2 (E2-FL) protein could elicit specific antibodies and the N-terminal and C-terminal regions epitopes provoked B and T cells responses using immunoinformatics studies [63]. In another study, E1 and E2 glycoproteins were expressed in Sf9 insect cells using the baculovirus expression system producing trimeric, glycosylated CHIKV spikes. The E2 CHIKV recombinant protein conjugated with Poly (I:C) adjuvant provoked the highest immune responses in both arms in C57BL/6 mice [14].

In this study, using comprehensive immunoinformatics approaches, we developed an MEV candidate that included HTL, B cell, and CTL epitopes which were selected from structural proteins (E1, E2, 6 K, and E3) of the CHIKV. Additionally, PADRE epitope and RS09 as TLR4 agonist were used as adjuvant in the designed the MEV construct. This adjuvant is sufficiently safe and able to provoke CTL epitopes [33]. We observed that the physicochemical traits of the MEV candidate were acceptable as evaluated by the ProtParam server. The molecular weight of the designed MEV construct included 14.795 kDa and similar to previous studies revealed appropriate antigenic characteristics [64–66], because vaccines with a molecular weight less than 5–10 KDa are supposed to be weak immunogens, whereas the pI value of 10 indicated the protein basic conditions in nature, and an instability index of 35.03 competent the MEV as a stable protein similar to designed vaccines in previous studies [64–68], and proteins with instability index <40 are stable. The aliphatic index was estimated at 84.67 demonstrating the MEV construct as a high thermostable protein according to previously designed MEV candidates [64–68]. A high aliphatic index is an indication of high thermostability of a protein. The GRAVY score was -0.326, a negative GRAVY value reflects hydrophilic nature of a protein which was similar to those scores in previous studies [64–68]. This is responsible for better interaction with the polar environment. Immune simulation results confirmed that our designed MEV was able to stimulate immune responses (cellular, humoral, and innate immune). The humoral immune simulation such as IgG1 + IgG2, IgM, and IgG + IgM antibodies production, the B cell population, and the cellular immune response, HTLs, memory and CTLs were enhanced as well as recently published work on an MEV candidate against the SARS-CoV-2 [64]. The NK (natural killer) and DCs activity was found to be consistent along with higher macrophage activity in our designed MEV but they were not checked in another MEV against the SARS-CoV-2 [64]. The elicitation of a suitable immune response was confirmed by high levels of IFN-γ and IL-2 production in the simulation; these results were consistent with a previous research related to a designed MEV to combat SARS-CoV-2 [68].

Immunogenic epitopes were selected from structural proteins of CHIKV in our MEV construct, while recently Safavi et al, selected T cell epitopes from non-structural proteins for designing an MEV against the SARS-CoV-2 [64]. For the humoral immune system provocation effectively via antibodies, the SARS-CoV-2 spike protein inducing domain was used in vaccine construct compared to our vaccine construct which selected B cell epitopes from structural proteins of the CHIKV. According to *in silico* evaluations, our designed MEV construct had higher antigenic properties (Vaxijen; 0.65 and Antigenpro; 0.8) compared to recently published research on an MEV candidate against SARS-CoV-2 [64]. Additionally, both the MEV constructs were predicted as non-allergen.

Moreover, the Ramachandran plot confirmed that 99.1 percent of residues were within the most favored or in-allowed regions. The docked MEV-TLR4 complex unraveled the stability interacted via several bonds. According to the RMSD plot, the MEV-TLR4 complex was more stable than the MEV construct free form. The RMSF inferred no unusual fluctuations in the neither free nor docked state of the MEV candidate construct. The Rg score and dssp indicated the stability of both structures after 65ns and negligible secondary structure alteration.

In a study by Narula et al, an MEV candidate was employed against the CHIKV *strain S27-African prototype* using various structural and non-structural proteins and the β-defensin as the adjuvant. T cells, INF-γ, and B cell epitopes were predicted against capsid, E1-E3, and Nsp1-Nsp4 proteins and demonstrated potential immunogenicity efficacy. The docking and MD simulation were performed with the TLR3 [69]. The recombinant E1 and E2 envelope proteins of CHIKV have been used to elicit Th1/Th2 (along with high levels of pro-and anti-inflammatory cytokines in splenocytes) and antibody responses in mice [21]. In a study among E1 sequences, NTQLSEAHVEKS epitope was highly conserved, safe and efficient, eliciting B cells. Moreover, the KTEFASAYR epitope was conserved and promising for the T cells provocation [16]. Common epitopes of CHIKV and Mayaro viruses could elicit B cells and T cells epitopes using *in silico* approaches with population coverage of 92.43% worldwide [20]. In an *in silico* and docking study, E1 and E2 potential epitopes respectively included “SEDVYANTQLVLQRP” and “IMLLYPDHPTLLSYR” eliciting B and T cells responses and also binding to the HLA-I and HLA-II molecules at extremely low IC50 value. These epitopes exhibited over 80–90% and 60–80% population coverage, respectively [17]. An immunoinformatics study using the NSP2-CHIKV introduced B cell inducing VVDTTGSTKPDPGD epitope and HLA-A-binding epitopes of QPTDHVVGEY, FSKPLVYY, SLSESATMVY, and VTAIVSSLHY and HLA-DRB*01:01 binding VVGEYLVLSPQTVLRS epitope which demonstrated promiscuity [18].

There are few studies regarding the development of MEV candidates against CHIKV to evaluate proteins and adjuvants and compare the results. This study methods were promising in terms of the employment of more suitable online servers and adjuvants which provided valid results and sufficient immune responses. As TLR4 is expressed onto various immune cell types (macrophages, monocytes, DCs, and granulocytes), related immunization will provide acceptable immune responses [70]. Additionally, our selection of universal viral sequence and polyprotein provoked more inclusive immunization. Our survey inferred that the designed CHIKV MEV construct using structural proteins (E1, E2, 6 K, and E3) epitopes can stimulate cellular and humoral immune responses. The main limitations of this study included the lack of experimental studies such as *in vitro*, *in vivo*, and clinical trials to verify the results.

## 5. Conclusion

CHIKV is an emerging viral infectious agent with the potential of causing a pandemic without any approved vaccine or therapeutic approach. In this study, using comprehensive immunoinformatics analyses, we developed a novel MEV candidate using CHIKV structural polyprotein (E1, E2, 6 K, and E3). The selected potential epitopes were immunogen which efficiently stimulated immune responses using the proper and safe synthetic adjuvant including PADRE and RS09 designed for the MEV construct. Immune provocation included predicted HTL, B cell, and CTL epitopes. The MEV *in silico* cloning was successful in *E*. *coli* host with a high level of expression. *In vitro*, *in vivo*, and clinical trial studies are required to verify the findings of the current study.

## Supporting information

S1 Fig The predicted 3D structure of the designed multi-epitope peptide vaccine by I-TASSER server. Dark green and orange: adjuvant, dark blue: helper T lymphocyte epitopes, red: B cell epitopes, blue: cytotoxic T lymphocyte epitopes.
(DOCX)

S2 Fig Hydrogen bond plot of vaccine-TLR4 complex. (DOCX)

S3 Fig Probability distribution of hydrogen bonds between vaccine-TLR4 complexes. (DOCX)

S4 Fig Percentage of secondary structure of vaccine in state and docked form. (DOCX)

S5 Fig Determination of the codon-optimized gene parameters in E.coli host. a. CAI of the sequence is 0.85, while a CAI >0.8 is rated as good for expression in the desired expression host. b. The average GC content of the sequence is 52.36%. The percentage GC content between 30–70% is proper. c. Codons with lower CFD value than 30 are likely to hamper the expression efficiency and here the percentage of low frequency (<30%) codons based on E.coli host organism is 0. (d) Minimal free energy (MFE) secondary structure and (e) Centroid secondary structure of the vaccine mRNA.
(DOCX)
