# Supplementary material for: In silico design and validation of a novel multi-epitope vaccine candidate against structural proteins of Chikungunya virus using comprehensive immunoinformatics analyses
Source: PLoS One. 2023 May 5;18(5):e0285177. doi: 10.1371/journal.pone.0285177 (PMC10162528; doi:10.1371/journal.pone.0285177)
Supplement: S1 Fig — Dark green and orange: adjuvant, dark blue: helper T lymphocyte epitopes, red: B cell epitopes, blue: cytotoxic T lymphocyte epitopes. (DOCX) [file pone.0285177.s001.docx]

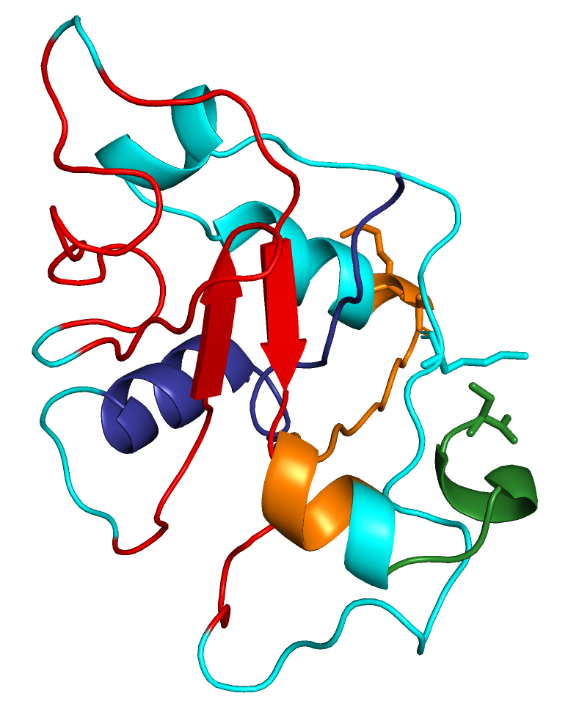


Fig S1. The predicted 3D structure of the designed multi-epitope peptide vaccine by I-TASSER server. Dark green and orange: adjuvant, dark blue: helper T lymphocyte epitopes, red: B cell epitopes, blue: cytotoxic T lymphocyte epitopes
