# Supplementary figures and images for: In silico design and validation of a novel multi-epitope vaccine candidate against structural proteins of Chikungunya virus using comprehensive immunoinformatics analyses
Source: PLoS One. 2023 May 5;18(5):e0285177. doi: 10.1371/journal.pone.0285177 (PMC10162528; doi:10.1371/journal.pone.0285177)

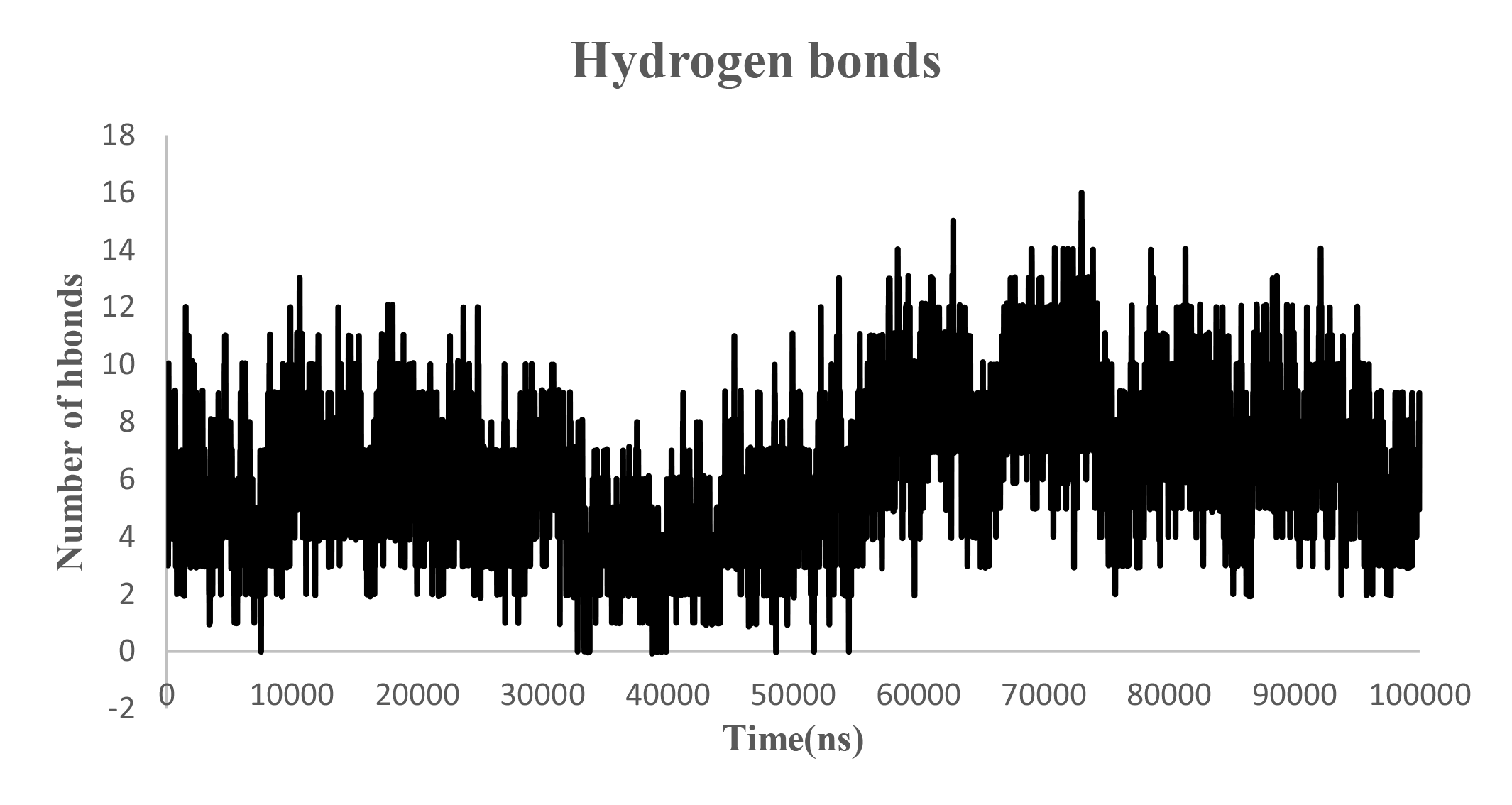


Fig S2. Hydrogen bond plot of vaccine-TLR4 complex

Supplement: S2 Fig — (DOCX) [file pone.0285177.s002.docx]

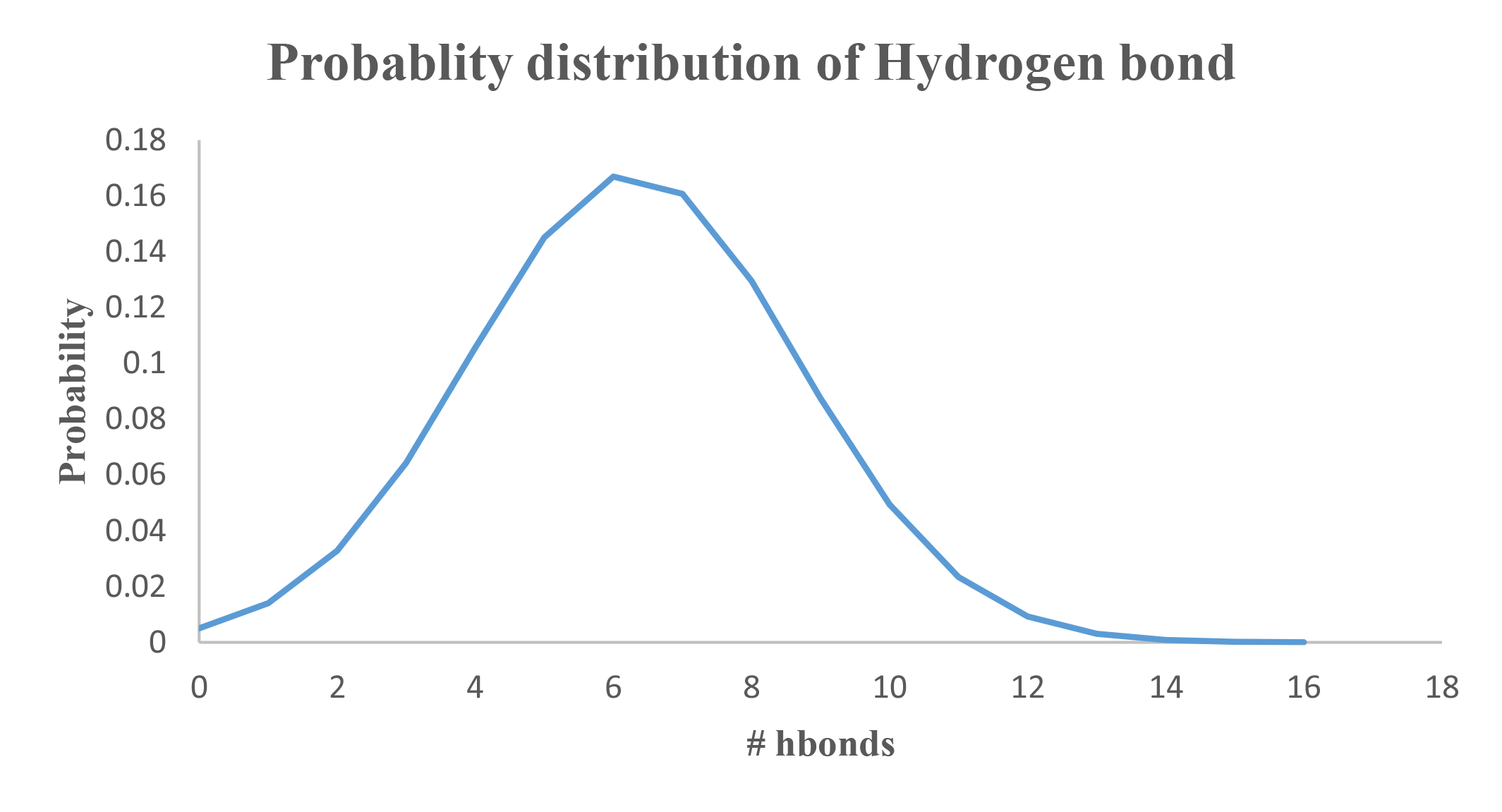


Fig S3. Probability distribution of hydrogen bonds between vaccine-TLR4 complexes

Supplement: S3 Fig — (DOCX) [file pone.0285177.s003.docx]

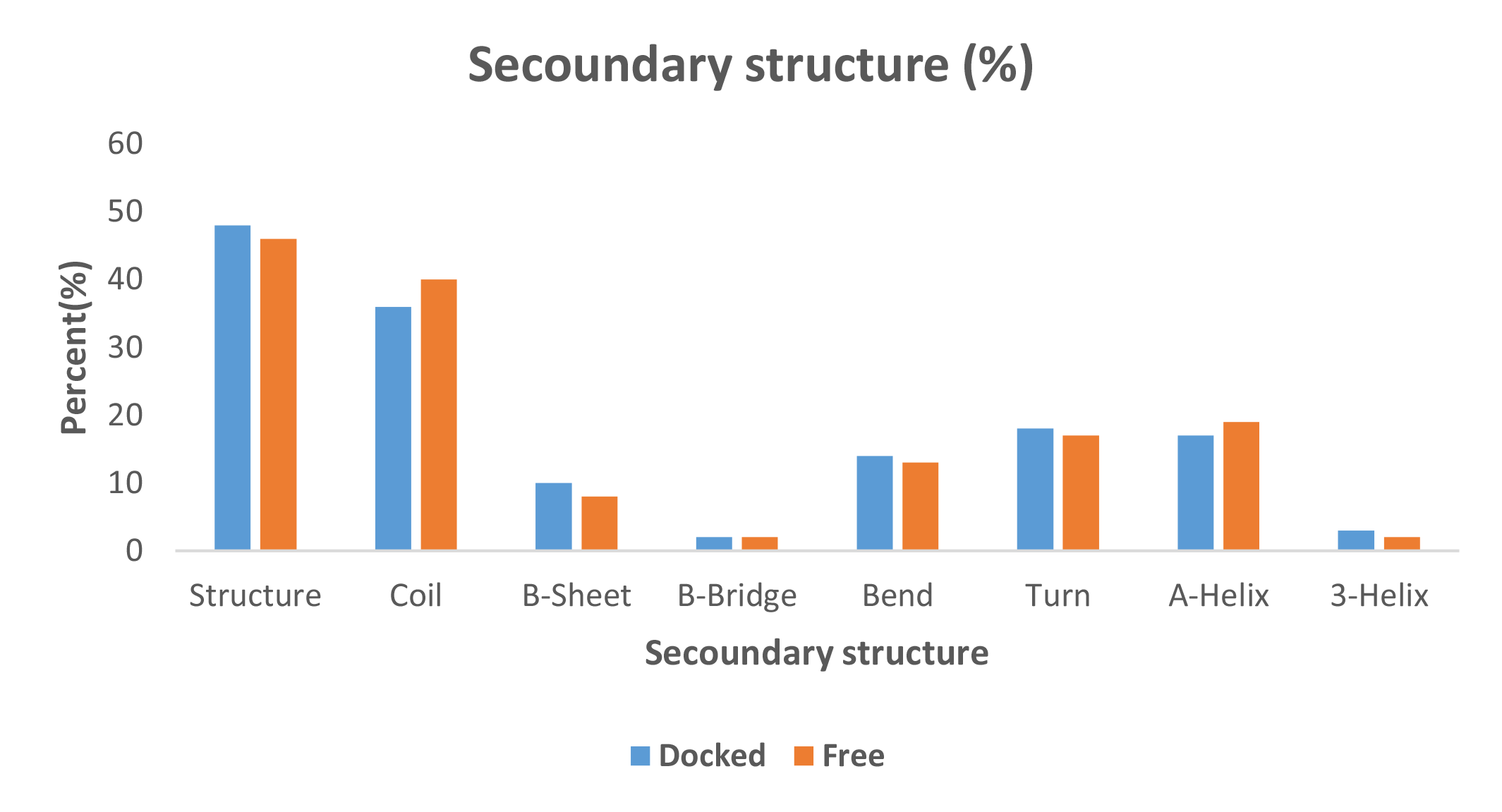


Fig S4. Percentage of Secondary structure of vaccine in state and docked form

Supplement: S4 Fig — (DOCX) [file pone.0285177.s004.docx]
