# Supplementary material for: In silico design and validation of a novel multi-epitope vaccine candidate against structural proteins of Chikungunya virus using comprehensive immunoinformatics analyses
Source: PLoS One. 2023 May 5;18(5):e0285177. doi: 10.1371/journal.pone.0285177 (PMC10162528; doi:10.1371/journal.pone.0285177)
Supplement: S5 Fig — a. CAI of the sequence is 0.85, while a CAI >0.8 is rated as good for expression in the desired expression host. b. The average GC content of the sequence is 52.36%. The percentage GC content between 30–70% is proper. c. Codons with lower CFD value than 30 are likely to hamper the expression efficiency and here the percentage of low frequency (<30%) codons based on E.coli host organism is 0. (d) Minimal free energy (MFE) secondary structure and (e) Centroid secondary structure of the vaccine mRNA. (DOCX) [file pone.0285177.s005.docx]

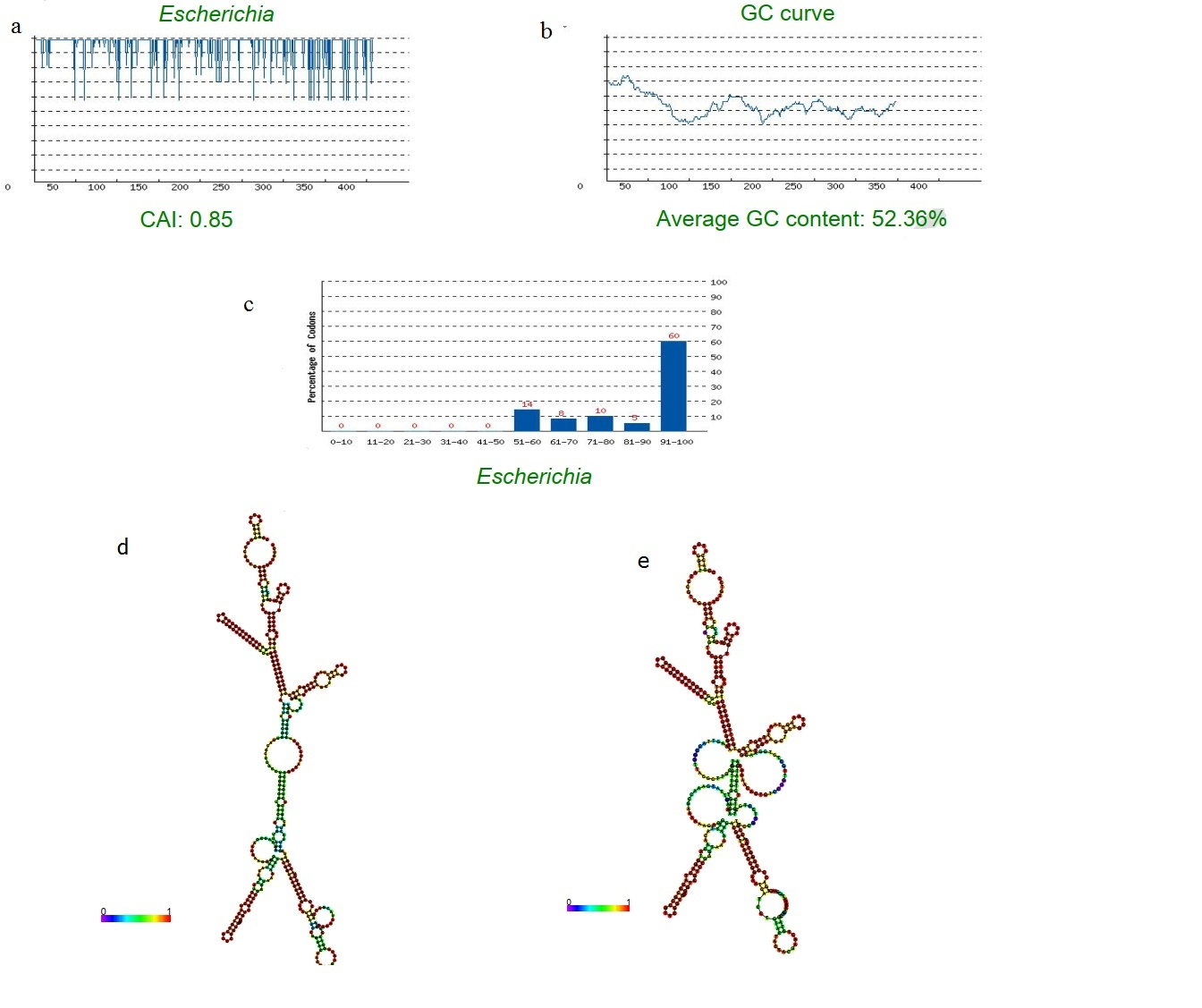


Fig S5. Determination of the codon-optimized gene parameters in *E.coli* host. a. CAI of the sequence is 0.85, while a CAI *>*0.8 is rated as good for expression in the desired expression host. b. The average GC content of the sequence is 52.36 %. The percentage GC content between 30-70% is proper. c. Codons with lower CFD value than 30 are likely to hamper the expression efficiency and here the percentage of low frequency (*<*30%) codons based on *E.coli* host organism is 0. (d) Minimal free energy (MFE) secondary structure and (e) Centroid secondary structure of the vaccine mRNA
